# Electrochemical analysis of Na–Ni bimetallic phosphate electrodes for supercapacitor applications

**DOI:** 10.1039/c9ra04487f

**Published:** 2019-08-12

**Authors:** Abdulmajid A. Mirghni, Kabir O. Oyedotun, O. Olaniyan, Badr A. Mahmoud, Ndeye Fatou Sylla, Ncholu Manyala

**Affiliations:** Department of Physics, Institute of Applied Materials, SARChI Chair in Carbon Technology and Materials, University of Pretoria Pretoria 0028 South Africa ncholu.manyala@up.ac.za +27 12 420 3549

## Abstract

Bimetallic sodium–nickel phosphate/graphene foam composite (NaNi_4_(PO_4_)_3_/GF) was successfully synthesized using a direct and simple precipitation method. The hierarchically structured composite material was observed to have demonstrated a synergistic effect between the conductive metallic cations and the graphene foam that made up the composite. The graphene served as a base-material for the growth of NaNi_4_(PO_4_)_3_ particles, resulting in highly conductive composite material as compared to the pristine material. The NaNi_4_(PO_4_)_3_/GF composite electrode measured in a 3-electrode system achieved a maximum specific capacity of 63.3 mA h g^−1^ at a specific current of 1 A g^−1^ in a wide potential range of 0.0–1.0 V using 2 M NaNO_3_ aqueous electrolyte. A designed and fabricated hybrid NaNi_4_(PO_4_)_3_/GF//AC device based on NaNi_4_(PO_4_)_3_/GF as positive electrode and activated carbon (AC) selected as a negative electrode could operate well in an extended cell potential of 2.0 V. As an assessment, the hybrid NaNi_4_(PO_4_)_3_/GF//AC device showed the highest energy and power densities of 19.5 W h kg^−1^ and 570 W kg^−1^, respectively at a specific current of 0.5 A g^−1^. The fabricated device could retain an 89% of its initial capacity with a coulombic efficiency of about 94% over 5000 cycling test, which suggests the material's potential for energy storage devices applications.

## Abstract 2

CV curve of the hybrid device as reflection of the conductive NaNi_4_(PO_4_)_3_/GF composite and AC.

## Graphical abstract

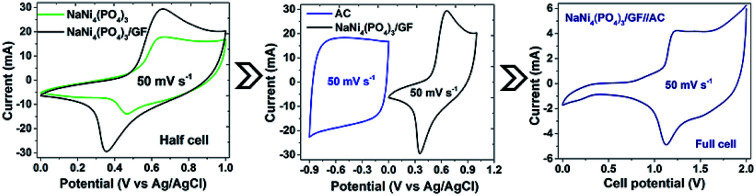

## 1. Introduction

With the advent of electric vehicles and portable electronic devices human demand for energy has continued to increase. Due to the energy consumption rate of these components, energy is regarded as the major problem facing humanity.^1,2^ Although there are different types of energy sources^3^ that could be explored to power these devices, the renewable sources (such as wind and solar) have been mooted as the best option to use due to the low or zero green gas emission rate. However, as a result of the intermittent nature of the green energy sources, their successful exploitation requires reliable and efficient electrical energy storage systems. Following this development, the design of new energy storage devices remains an active and expanding field of research.^4,5^ Electrochemical energy storage systems, which include but are not limited to batteries and electrochemical capacitors (ECs),^6^ have been embraced as efficient storage devices. The reason for this is that the storage technique takes advantage of the fact that both chemical and electrical energy have a common charge carrier (known as the electron), thus limiting losses due to energy conversion from one form to another. For many years, batteries have been used as the storage devices mainly because of their high energy density. However, they take time to charge and discharge, which reduces their tendency to deliver power. In applications requiring high and instant power, engineering the right battery for the task would typically lead to an increase in the size and cost requirements. Consequently, supercapacitors are very attractive candidates in the field of energy-storage to replace conventional chemical batteries for use in hybrid vehicles, portable electronics and backup energy systems owing to the strength of their unique power densities, fast charge–discharge rate, and stable cycling performance.^7,8^ Supercapacitors are generally classified into two categories based on the storage mechanism: the electrical double layer capacitors (EDLC) (by electrostatic storage) and the pseudocapacitors (by fast redox process), which can overcome the low energy density of EDLC.^9^ However, the shortage that restricts the application of supercapacitors in portable electronic devices is the design and also the bulkiness of the device. So the requirements of such devices like mobile phones and wearable electronics are lightweight, safety, flexibility, high-performance and reliable power sources.^10^

As a sort of charge mechanism storage, the performance of supercapacitors configuration depends on the fundamental characteristics of the materials in the cell. Transition metal oxide as well as conducting polymer has been strongly considered as active materials for supercapacitors. Presently, due to excellent chemical stability, rich valences, and good electrochemical performance, various transition metal oxides have also been candidates in this field.^11–14^ Among these transition metal oxides (TMOs), such as RuO_2_ and IrO_2_, RuO_2_ is a conductive metal oxide, with three oxidation states accessible within 1.4 V, exhibiting an impressive reported specific capacitance up to 2000 F g^−1^.^15^ RuO_2_ is known to be the most effective electrode material, but the extremely high cost and toxicity limit its applications.^16^ Hence, cheaper transition metal oxides, such as nickel oxides, cobalt oxides, manganese oxides and iron oxides, are actively studied and found to exhibit good electrochemical performance. This has triggered strong research interest in the materials. Among these, nickel phosphates are regarded as good candidates owing to their low cost, lower toxicity, easy synthesis and high theoretical specific capacity, while sodium phosphate is a novel energy storage cathode material based on an affordable and globally available element.^17,18^

Besides the oxides, transition metal phosphates, on the other hand, have reported performing in a promising manner when subjected to the test of safety, environmental inertness, structural stability and energy efficiency.^19^ Li-based phosphates having a common formula LiMPO_4_ (M = Ni, Fe, Co or Mn) have been extensively used as a cathode material for a battery. These compounds contain tetrahedral anion structure units (XO_4_)^*n*−^ with strong covalent bonding, generating oxygen octahedral sites occupied by other metal ions.^20^ Among the several metal atom substitutions, lithium–iron and lithium–manganese phosphates have been observed as promising cations in the olivine structure. This is mainly due to low cost, environmental inertness, cycling stability and a high degree of reversibility.^21^ Na-based phosphate with chemical formula NaMPO_4_ (M = Mn, Co, Ni and Fe) is also considered as an interesting cathode candidate for the new generation of sodium-ion batteries.^22^ This consideration is mainly owing to some unique advantages including thermal stability and higher voltage due to the inductive effect which made the phosphate-based materials to be the capable cathode materials in Na-ion batteries.^23^

To the best of knowledge, there are insufficient studies reported considering metal phosphate as an electrode for supercapacitor application. Zhang *et al.* reported the synthesis of a series of mesoporous Ni_*x*_Co_3−*x*_(PO_4_)_2_ hollow shells where they have used boiling a mixture at 185 °C. The Ni_*x*_Co_3−*x*_(PO_4_)_2_ hollow shells has used as a material for supercapacitor, sensor and catalyst applications. As a supercapacitor electrode, the material achieved a specific capacitance of 940 F g^−1^ at a specific current of 1 A g^−1^ and 85% of the initial capacitance was maintained after 1000 cycles.^24^ Senthilkumar *et al.* reported the synthesis of NaMPO_4_ (M = Mn, Co and Ni) maricite-type following a solution combustion method. The materials have tested in 1 M NaOH and achieved specific capacitance as follow: NaMnPO_4_ (163 F g^−1^), NaCoPO_4_ (249 F g^−1^) and NaNiPO_4_ (368 F g^−1^). Moreover, the NaNiPO_4_ maricite-type used as positive electrode in a device with AC as a negative electrode, where energy density of 20 W h kg^−1^ and power density of 138 W kg^−1^ were reported.^25^ Ha-Kyung Roh *et al.* synthesized NaTi_2_(PO_4_)_3_/rGO through direct precipitation of NaTi_2_(PO_4_)_3_ nano-sized on rGO. 1 M NaClO_4_ dissolved in a mixture of ethylene carbonate (EC) & dimethyl carbonate (DMC), (1 : 1) was used as an electrolyte. The NaTi_2_(PO_4_)_3_/rGO was used as a negative electrode in a device with AC as a positive electrode. The device could operate in a wider voltage of 2.7 V as result of organic electrolyte used. Consequently, an energy density of 53 W h kg^−1^ and a power density of 334 W kg^−1^ at specific current of 0.25 A g^−1^ was delivered.^26^ On the other hand, in order to efficiently enhance the performance of monometallic active material (for example NiPO_4_); one can take advantage of the synergistic effect of combining two metals in a bimetallic active material. A bimetallic phosphate *e.g.*, NaNiPO_4_ in contrarily to the monometallic phosphate is expected to provide a better redox process. Firstly, we are using the highly conductive Ni cation in sodium analogue in an aqueous electrolyte.^25^ Secondly, an addition of graphene into the matrices of the material is another practical step of compositing. Graphene is considered to be motivating choice for composite-material. Graphene has an attractive advantage like high electronic conductivity (16 000 S m^−1^), high intrinsic carrier mobility (200 000 cm^2^ V^−1^ s^−1^), high specific surface area (2630 m^2^ g^−1^, theoretical value), and high superior mechanical strength which makes it a favorable material in a wide diversity of applications.^27^ Furthermore, flexible graphene also serves as a buffer to alleviate possible volume changes during charge/discharge process, especially in cyclability test processes.^28,29^

In the present work, we introduced a direct and easy precipitation method to synthesize a novel hierarchical nanosphere bimetallic sodium–nickel phosphate and demonstrated its capacitive capability in aqueous electrolyte. These distinctive nanospheres brought out the synergy of conductive Ni cation in sodium analogue composited with a conductive graphene. A comparison of pristine NaNi_4_(PO_4_)_3_ and composite NaNi_4_(PO_4_)_3_/GF as electrodes for supercapacitors in 3 electrode configuration aqueous electrolyte were carried out. The composites NaNi_4_(PO_4_)_3_/GF has resulted in a superior capacitive properties than the pristine NaNi_4_(PO_4_)_3_ in a wide potential window up to 1.0 V using a 2 M NaNO_3_ aqueous electrolyte. The composite NaNi_4_(PO_4_)_3_/GF was then used as a positive electrode in a hybrid cell device with activated (AC) as a negative electrode. The hybrid NaNi_4_(PO_4_)_3_/GF//AC was found to operate in a very wide cell potential of 2.0 V.

## 2. Materials and method

### 2.1. Materials

Nickel nitrate hexahydrate (Ni(NO_3_)_2_·H_2_O), sodium nitrate (NaNO_3_), ammonium phosphate (N_2_H_9_PO_4_) and urea (CH_4_N_2_O) were purchased from Sigma Aldrich and used as received without further modification. Graphene foam used in this work was prepared by means of an atmospheric pressure chemical vapor deposition (APCVD) technique as reported in our previous work.^30^

### 2.2 Synthesis of NaNi_4_(PO_4_)_3_/GF

**Scheme 1:**
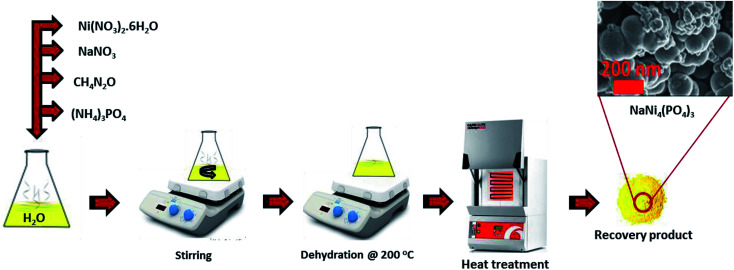
Synthesis steps of nanostructured NaNi_4_(PO_4_)_3_.

For the synthesis of pristine NaNi_4_(PO_4_)_3_, nickel nitrate hexahydrate (5.82 g), sodium nitrate (1.70 g), ammonium phosphate (2.64 g) and urea (1.20 g as a reducing agent) were all dissolved in 20 ml deionized water and stirred for 30 min. The mixture was then placed on a hot plate at 200 °C for dehydration. After complete dehydration the obtained yellowish solid was then transferred into an atmospheric electric furnace set at 300 °C for 15 min for heat treatment as shown in Scheme 1. The same procedure was followed for the synthesis of the NaNi_4_(PO_4_)_3_/GF composite but with addition of 60 mg graphene foam (GF).

### 2.3 Synthesis of activated carbon (AC)

The AC sample used in this work was prepared in two steps as follows: initially, the peanut shell (PS) was washed with deionized (DI) water and dried in ambient condition before being ground to fine powder. Afterward, 5 g of the powder (PS), 1 g of NaCl and 0.5 g urea were mixed together with DI water to form a mud cake, which was placed in an electric oven at 80 °C overnight to dry. The prepared cake was then carbonized at 600 °C under argon gas for 2 h at 5 °C per min heating rate to obtain a hydrochar. The recovered hydrochar was washed several times with DI water and dried in an oven for 12 h at 80 °C. Thereafter, the hydrochar was mixed with KOH in a ratio of 1 : 4 and then transferred into a horizontal tube furnace for activation at 850 °C for 1 h at a ramping rate of 5 °C per min under Ar gas. The final product was soaked in 3 M HCl to remove residual KOH, and then washed with DI water until neutral pH was obtained and then dried at 80 °C overnight.

### 2.4 Structural characterization

X-ray diffraction was used for structure analysis in the range 2 theta = 15–80°. The specification of the instrument is as follow: XPERT-PRO diffractometer (Analytical BV, the Netherlands) with *θ*/2*θ* geometry and a counting time of 5.240 s per step. Qualitative phase examination of the samples was directed with the X'Pert Highscore search-match software at room temperature using Co K1α (*λ* = 0.178897 nm). Surface area was obtained by the Brunauer–Emmett–Teller (BET) technique from the N_2_ adsorption isotherm technique from a Micrometrics Tristar II 3020 (version 2.00) Analyser at 90 °C. The pore size distribution plot was obtained from the desorption branch of the Barrett–Joyner–Halenda (BJH) plots. The SEM images were obtained on a Zeiss Ultra Plus 55 field emission scanning electron microscope (FE-SEM) (Carl Zeiss, Oberkochen, Germany) operated at an accelerating voltage of 2.0 kV. The Raman spectra for the samples were obtained using a WITec alpha 300 RAS+ confocal micro-Raman microscope (Focus Innovations, Germany) at a 532 nm laser wavelength was adopted to characterize the samples with a spectral acquisition time of 150 s and laser power of 5 mW.

### 2.5 Electrochemical characterization

To make electrodes for 3-electrode configuration, 80% of the active material was mixed with 10% carbon black and 10% polyvinylidene difluoride (PVDF) as a binder. The role of the carbon black on the mixture was to increase the conductivity of the material. A 1-methyl-2-pyrrolidinone (NMP) was later added to make slurry. After perfect mixture of the material with the NMP, pasted on the nickel foam with a diameter of 1 cm^2^ and allocated in an oven set in 70° overnight to evaporate the NMP. The electrochemical measurements were done in a 3-electrode system using Ag/AgCl (3 M KCl saturated) and glassy carbon as the reference electrode and counter electrode respectively. A neutral 2.0 M NaNo_3_ aqueous electrolyte was used in this experiment. A coin cell type CR2025 was used to assemble the hybrid supercapacitor. Different anodic and cathodic materials were used and the microfiber glass filter paper used to separate the two electrodes. Cyclic voltammetry (CV) was applied to confirm the cell-type while galvanostatic charge–discharge (GCD) was applied for further confirmation of the cell-type and also to calculate the cell capacity. Both the CV and CD were carried out in a cell potential ranging between 0.0 and 2 V. The impedance of the hybrid was also investigated with electrochemical impedance spectroscopy (EIS) in range 100 kHz to 0.01 Hz with zero potential amplitude using a Bio-logic VMP-300 potentiostat (Knoxville, US) driven by the EC-lab software.

## 3. Results and discussion

### 3.1 Structure, texture and morphology

**Fig. 1:**
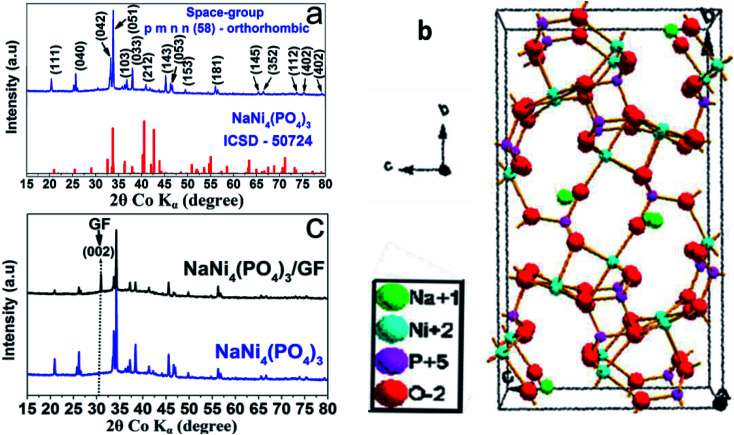
(a) XRD patterns of the as-prepared samples with NaNi_4_(PO_4_)_3_ (ICSD no. 50724), (b) a possible crystal structure of NaNi_4_(PO_4_)_3_ obtained using ICSD card no. 50724 and (c) the XRD pattern of as-prepared NaNi_4_(PO_4_)_3_ and NaNi_4_(PO_4_)_3_/GF composite.

The crystal structure of the as-prepared NaNi_4_(PO_4_)_3_ nano-spheres was characterized by X-ray diffraction (XRD) using Diamond software.^31^ In Fig. 1(a) the red vertical lines indicate the peak positions of standard NaNi_4_(PO_4_)_3_ (ICSD – 50724), space group: *pmnn* (58) orthorhombic. From Fig. 1(a), all of the diffraction peaks are sharp and can be well indexed with NaNi_4_(PO_4_)_3_ (ICSD – 50724). These diffraction peaks with narrow shapes indicate that the sample possesses good crystallinity. Usually, crystalline materials can electrochemically perform better since their stable crystal structure cannot be destroyed easily during charge–discharge processes.^32^Fig. 1(b) shows the possible schematic crystal structure of the sample. The information from the standard PDF card reflects the presence of strong electron localization in NaNi_4_(PO_4_)_3_. Moreover, altering the site of Na and Ni causes the triphylite form creating more space for Na ions to occupy and intercalate along one-dimensional channel in its orthorhombic framework, which is an essential factor for energy storage host materials. Fig. 1(c) shows the XRD pattern of the NaNi_4_(PO_4_)_3_ and NaNi_4_(PO_4_)_3_/GF composite. It can be observed that with an introduction of GF the XRD pattern of the NaNi_4_(PO_4_)_3_/GF/GF remained unchanged however, a peak corresponding to GF was observed at about 30.9 degree and indexed as (002). This confirms the presence of GF in the NaNi_4_(PO_4_)_3_/GF composite.

**Fig. 2:**
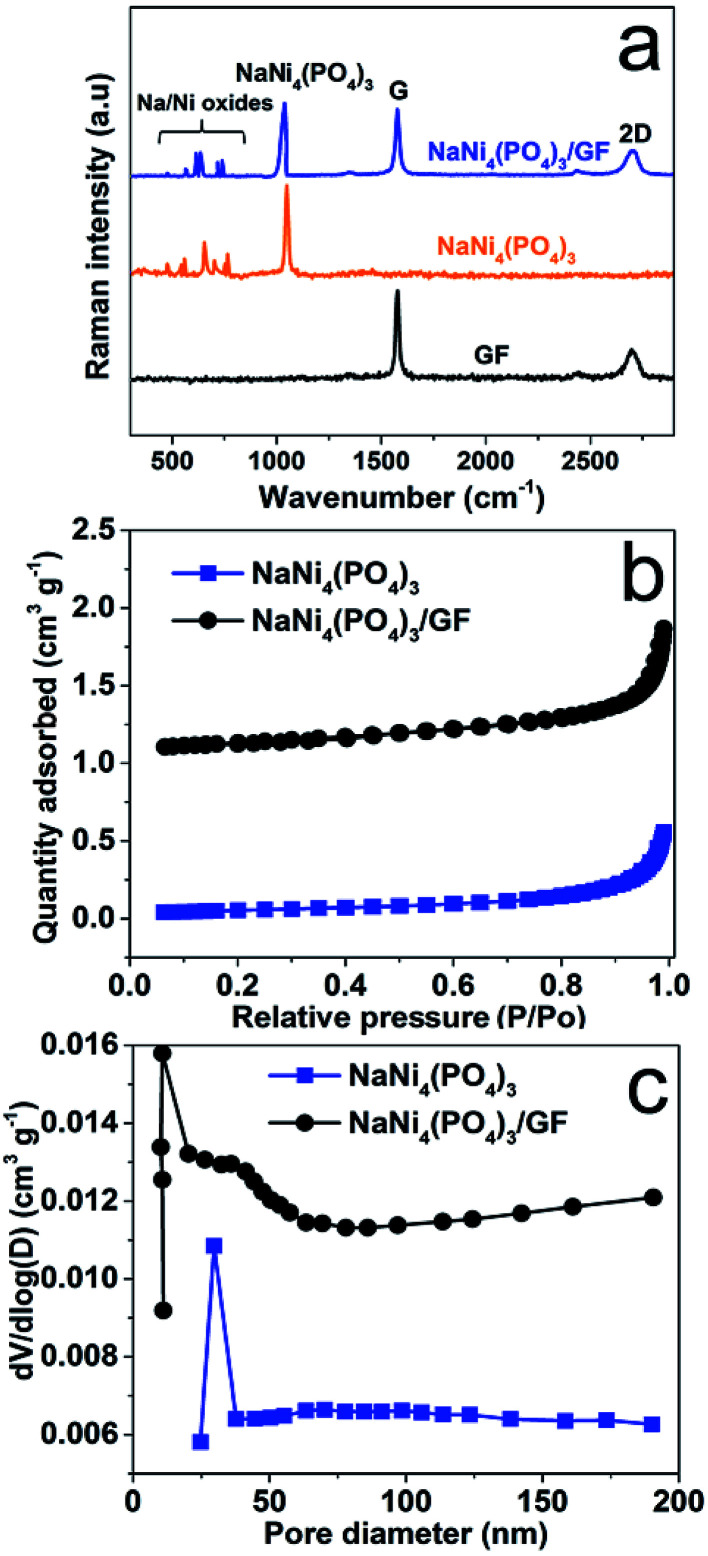
(a) Raman spectrum of GF, NaNi_4_(PO_4_)_3_ and NaNi_4_(PO_4_)_3_/GF, (b) N_2_ adsorption/desorption isotherms of pristine NaNi_4_(PO_4_)_3_ and NaNi_4_(PO_4_)_3_/GF composite and (c) the pore size distribution of pristine NaNi_4_(PO_4_)_3_ and NaNi_4_(PO_4_)_3_/GF composite.

Fig. 2(a) shows Raman analysis of the pure graphene foam (GF), NaNi_4_(PO_4_)_3_, and NaNi_4_(PO_4_)_3_/GF composite. G and 2D band were observed at about 1577 cm^−1^ and 2701 cm^−1^ respectively as Raman signature of graphene foam which are representative of C–C vibration mode and double resonance process.^33^ The G and 2D bands were also seen in NaNi_4_(PO_4_)_3_/GF but with slightly less intensity which confirms the existence of GF in this composite and also the incorporation of GF into the matrix of NaNi_4_(PO_4_)_3_ electrode material. The peak at about 1043 cm^−1^ on the NaNi_4_(PO_4_)_3_ as well as NaNi_4_(PO_4_)_3_/GF is attributed to the symmetric stretching and vibrational mode of PO_4_^3−^.^34^ Another Raman peaks were observed at about 737, 721, 636, 613, 566 and 473 cm^−1^ which could be referred to Na–Ni–O.^35,36^*I*_2D_/*I*_G_ peak ratio is calculated to be equal to ∼0.97 which is an indication that GF used to form the composite is a few layers.^34^ The pore texture of the NaNi_4_(PO_4_)_3_ and NaNi_4_(PO_4_)_3_/GF was analyzed by N_2_ adsorption/desorption measurements. Fig. 2(b) shows that the materials exhibit a type-III behavior with type-H3 hysteresis loop suggesting material containing mainly mesopores and macropores. The specific surface area (SSA) of the NaNi_4_(PO_4_)_3_ and NaNi_4_(PO_4_)_3_/GF are 4.3 m^2^ g^−1^ and 10.1 cm^2^ g^−1^ respectively. The increase in the specific surface area in the composite can be attributed to the presence of graphene foam. Consequently, the electrochemical performance of the composite is expected to be significantly improved. The pore size distributions (Fig. 2(c)) was calculated using the Barrett–Joyner–Halenda (BJH) analysis from the desorption branch and the pore size in the materials is mainly distributed within 5–50 nm range.

**Fig. 3:**
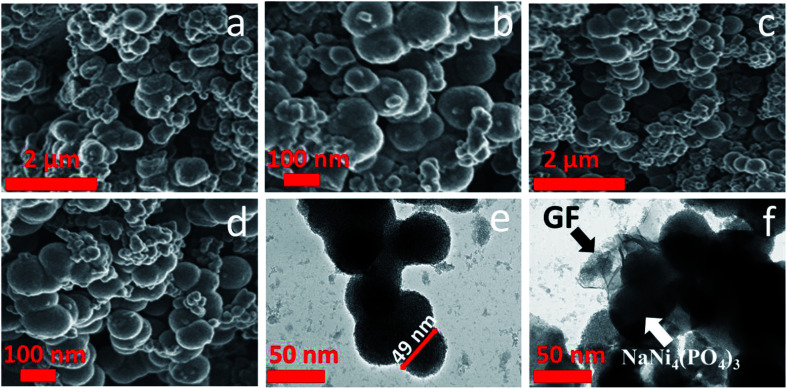
(a and b) SEM micrographs of pristine NaNi_4_(PO_4_)_3_ sample at low and high magnification, (c and d) NaNi_4_(PO_4_)_3_/GF composite at low and high magnification and (e and f) TEM images of pristine NaNi_4_(PO_4_)_3_ and NaNi_4_(PO_4_)_3_/GF composite.

Scanning electron microscopy (SEM) was used to investigate the morphology of the pristine and composite materials. Fig. 3(a) and (b) shows a low and high magnification SEM micrographs of pristine NaNi_4_(PO_4_)_3_ sample. They exhibit a hierarchical nano-spheres morphology and it can be seen that the NaNi_4_(PO_4_)_3_ nano-spheres are inter-connected with each other but still keeping a sphere-like morphology. Fig. 3(c) and (d) shows a low and high magnification of NaNi_4_(PO_4_)_3_/GF composite. It is worth mentioning that the graphene foam (GF) sheet could not be identified in the SEM morphology of the composites. This could be due to ultra-sonication of the graphene for hours after been dispersed in deionized water and also after the precursors were added to it.^37^

Further microscopic analysis of both the pristine NaNi_4_(PO_4_)_3_ and NaNi_4_(PO_4_)_3_/GF composite materials was investigated by using a transmission electron microscopy (TEM) technique, and is shown in Fig. 3(e and f). It can be clearly observed that the NaNi_4_(PO_4_)_3_ material composed of hierarchical spheres-like morphology in agreement with SEM images with a single sphere having an average diameter of about 49 nm, as shown in Fig. 3(e). A uniform dispersion of the NaNi_4_(PO_4_)_3_ nano-spheres wrapped with the graphene foam sheet was observed as shown in Fig. 3(f) which is needed for providing the necessary surface required for efficient charge transport and storage.

**Fig. 4:**
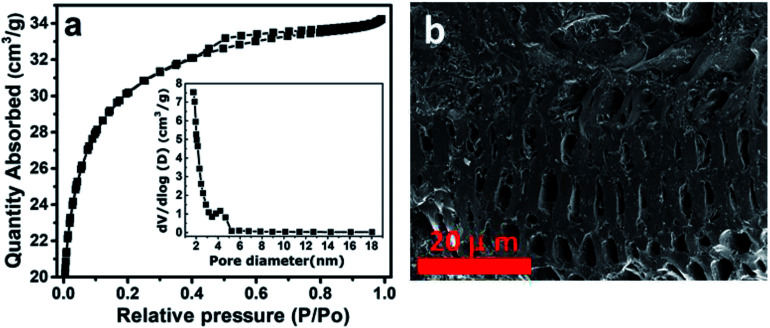
(a) BET analysis of activated carbon from the peanut shell (AC) and (b) SEM image of activated carbon from the peanut shell (AC).

Fig. 4(a) displays BET results of the activated carbon from the peanut shell (AC) used in this work. This figure shows type-IV and H4 hysteresis loop indicating the material possessing mainly mesopores and micropores as can be observed from the inset to the figure. The specific surface area obtained for this material was 2547 m^2^ g^−1^, suggesting the material contains a high surface area. The morphology of the activated carbon (AC) was determined by SEM. Fig. 4(b) represent SEM of the AC in 20 micrometer scale, and as can be seen, the material is full of cavities which are beneficial for the energy storage application.^38^

### 3.2 Electrochemistry (3-electrode measurements)

**Fig. 5:**
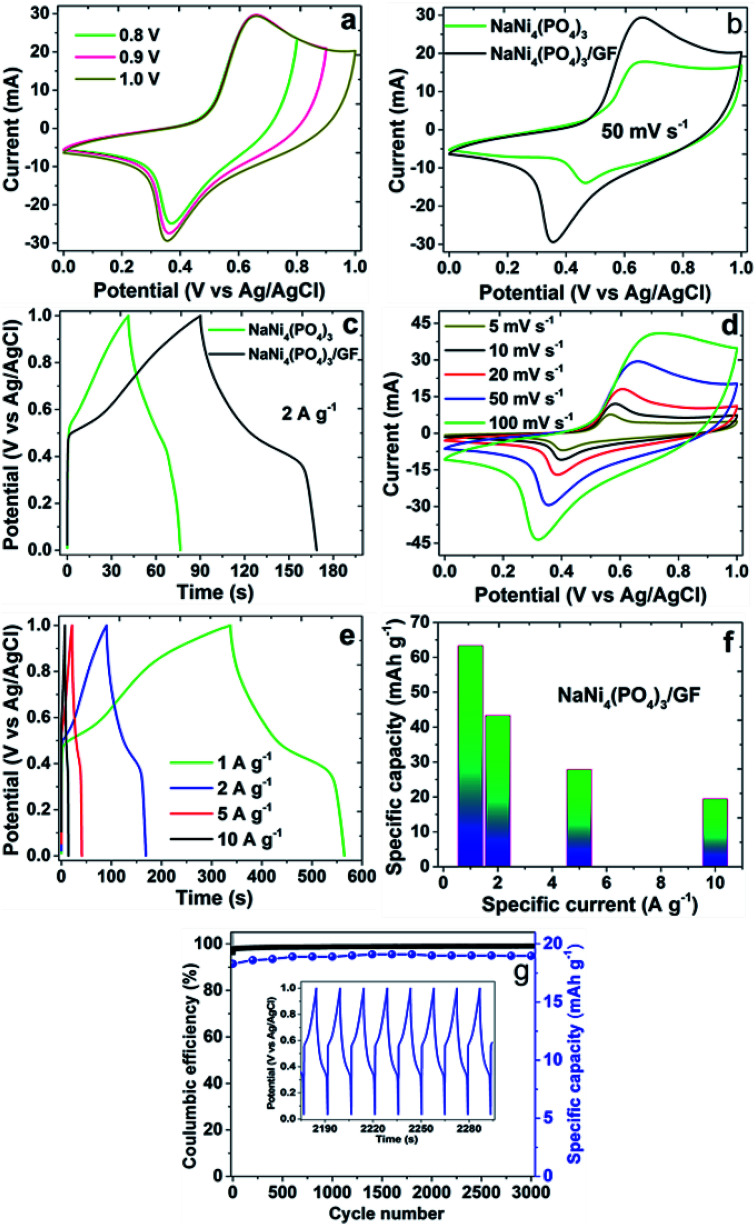
(a) CVs of NaNi_4_(PO_4_)_3_/GF composite at a different potential window at a scan rate of 50 mV s^−1^, (b and c) CV and GCD curves of pristine NaNi_4_(PO_4_)_3_ and NaNi_4_(PO_4_)_3_/GF composites at scan rate of 50 mV s^−1^ and specific current of 2 A g^−1^ respectively, (d and e) CV and GCD curves of NaNi_4_(PO_4_)_3_/GF composite at different scan rates and different specific currents respectively, (f) a specific capacity calculated for NaNi_4_(PO_4_)_3_/GF at different specific currents and (g) double *Y* plot illustrating coulombic efficiency and specific capacity *versus* cycle number of NaNi_4_(PO_4_)_3_/GF at a specific current of 10 A g^−1^ (the inset is some GCD cycles).

The performance of NaNi_4_(PO_4_)_3_ and NaNi_4_(PO_4_)_3_/GF composite hierarchical nanospheres as electrode materials for supercapacitors was investigated by cyclic voltammetry (CV), galvanostatic charge–discharge techniques (GCD) and electrochemical impedance spectroscopy (EIS). The experiment was performed in a 2 M NaNO_3_ electrolyte within a potential window range of 0.0–1.0 V. Fig. 5(a) represents CV curves of the NaNi_4_(PO_4_)_3_/GF investigated at different working potential windows ranging from 0.8 to 1.0 V in a 2 M NaNO_3_ neutral electrolyte. In these CVs an optimization to determine the stable potential window of the material was done. The CV curves exhibit redox reactions within these potential windows which is typical of faradaic supercapacitors signature. With increasing voltage range the CV still sustains the same shape with a slight increase in capacity value. The extended potential window of the material within 1.0 V could be due to the low concentration of hydrogen ions relative to those of acidic and basic electrolytes.^39^

Fig. 5(b) and (c) show a possible comparison of CV and GCD curves for pristine NaNi_4_(PO_4_)_3_ and composite NaNi_4_(PO_4_)_3_/GF at a scan rate of 50 mV s^−1^ and specific current of 2 A g^−1^ respectively. The shapes of the curves in these figures are different from those of ideal electrochemical double-layer capacitance, showing two well-defined redox peaks which are responsible for the faradaic behavior of the electrode materials. However, the current response and discharge time of CV and GCD curves respectively in NaNi_4_(PO_4_)_3_/GF composite are much higher than that of NaNi_4_(PO_4_)_3_ which is an indication that the NaNi_4_(PO_4_)_3_/GF electrode has a better capacitive performance than NaNi_4_(PO_4_)_3_. This can be attributed to the effect graphene foam has in the composite which had improved specific surface area as compared to the pristine sample indicated by BET results (see Fig. 2(a)).

Fig. 5(d) shows the CV curves of the NaNi_4_(PO_4_)_3_/GF composite at different scan rates. It can be seen that the current response of the CV curves increases gradually with increasing scan rate confirming the fast charge movement mechanism of the electrodes.^40^ The shape of the CV curves clearly implies that faradaic reactions occurred during the processes. The material exhibited a pair of redox peaks at around 0.3 V and 0.7 V which is due to the diffusion-controlled reversible redox reaction of Ni^2+^ ↔ Ni^3+^.^18^Fig. 5(e) shows the GCD curves of NaNi_4_(PO_4_)_3_/GF at different current densities in range of 1–10 A g^−1^. Visibly, the GCD curves are nonlinear which further confirms the faradaic property of the electrode material. Fig. 5(g) demonstrates cyclic stability of NaNi_4_(PO_4_)_3_/GF composite which was conducted under a constant GCD at 10 A g^−1^ for 3000 cycles. A 99% coulombic efficiency and 96% of its initial capacity retained revealing good electrochemical stability of the electrode material.

For the fact that the material shows nonlinear CV curves, a specific capacities were calculated for NaNi_4_(PO_4_)_3_/GF composite and found to be 63.3, 43.3, 27.8 and 19.4 mA h g^−1^ at specific currents of 1, 2, 5 and 10 A g^−1^ respectively (Fig. 5(f)). These specific capacities of the single electrode were calculated according to the following equation:^41^1
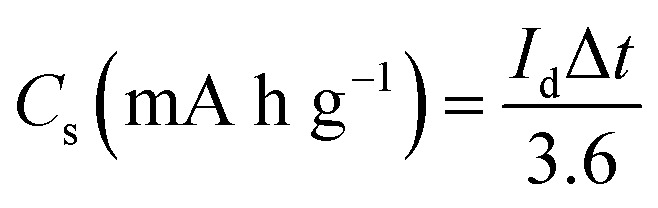
where *I*_d_ represents specific current (A g^−1^) and Δ*t* represents discharge time (s).

### 3.3 Electrochemistry (2-electrode measurements)

**Fig. 6:**
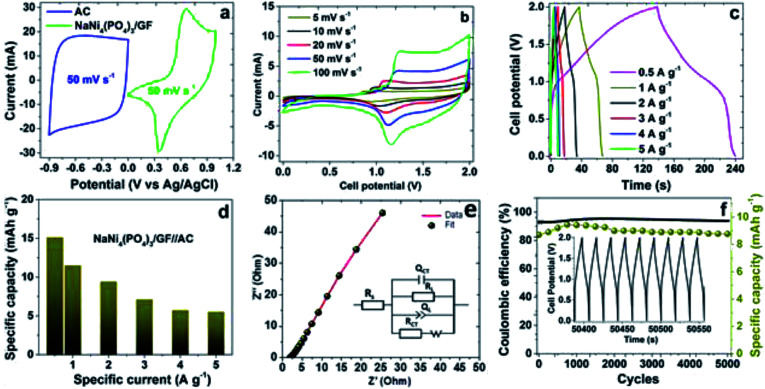
(a) A hybrid device demonstrating CV of NaNi_4_(PO_4_)_3_/GF as a positive electrode and AC as a negative electrode, (b) CV of the hybrid NaNi_4_(PO_4_)_3_/GF//AC measured at different scan rates, (c) GCDs of the hybrid NaNi_4_(PO_4_)_3_/GF//AC measured at different specific currents, (d) a specific capacity of hybrid NaNi_4_(PO_4_)_3_/GF//AC calculated at different specific current, (e) EIS of NaNi_4_(PO_4_)_3_/GF//AC (the inset is an equivalent circuit used to fit the EIS data) and (f) double *Y* plot showing coulombic efficiency and specific capacity *versus* cycle number for NaNi_4_(PO_4_)_3_/GF//AC hybrid device at specific current of 5 A g^−1^.

The NaNi_4_(PO_4_)_3_/GF was further used for the fabrication of hybrid device, a hybrid device was designed considering NaNi_4_(PO_4_)_3_/GF composite as a positive electrode material and AC as a negative electrode material as shown in Fig. 6(a). To fill the gap of the different current response as well as the different potential window between NaNi_4_(PO_4_)_3_/GF and AC, a charge balance was done using the formula (2) and (3). The hybrid device was assembled based on the charge stored in both positive and negative electrode ruled by the following formula:

2 *Q*_+_ = *Q*_−_

3 *m*_+_*I*_d(+)_Δ*t*_+_ = *m*_−_*I*_d(−)_Δ*t*_−_

Fig. 6(b) represent the CVs of the hybrid device NaNi_4_(PO_4_)_3_/GF//AC tested in scan rates of 5, 10, 20, 50, 100 mV s^−1^ within a cell potential range of 0.0–2.0 V. The curves portray two different characteristics, EDLC signature at about 0.0–1.0 V and redox faradaic reaction at about 1.0–2.0 V as a reflection of AC carbon and the metal phosphate materials. However, overall the curves depict a faradaic characteristic nature as the full cell potential considered. There are two defined peaks arisen in the curves as can be seen in Fig. 6(b) which are mainly subjected to the reversible redox reaction of Ni^2+^ ↔ Ni^3+^.^18^Fig. 6(c) represents GCD of the hybrid device NaNi_4_(PO_4_)_3_/GF//AC measured at specific currents of 0.5, 1.0, 2.0, 3.0, 4.0 and 5.0 A g^−1^ within a cell potential range of 0.0–2.0 V. The nonlinear GCD observed was typically characteristic of faradaic nature of the materials in agreement with CVs in Fig. 6(b). The specific capacity of the device (see Fig. 6(d)) at different specific currents of 0.5, 1.0, 2.0, 3.0, 4.0 and 5.0 A g^−1^ were calculated using eqn (1) to be 15.1, 11.2, 9.4, 7.1, 5.7 and 5.5 mhA g^−1^ respectively. These low values of the specific capacities are ascribed to the fact that neutral electrolytes have less ionic conductivity and bigger ionic hydrated radius compared to alkaline and acidic electrolytes.^42^

To study the conductivity and the ion diffusion control rate of the hybrid NaNi_4_(PO_4_)_3_/GF//AC, electrochemical impedance spectroscopy (EIS) measurement was conducted, the results of which are shown in Fig. 6(e) with the inset showing an equivalent circuit fitted with a ZFIT software associated with the BioLogic EC-lab.^43^ The equivalent circuit composed of the series resistance (*R*_s_), which represents the intrinsic resistance property of the electrode materials, electrolyte and the contact resistance between the electrode and the current collector interface. Charge transfer resistance *R*_CT_ and the interface connection capacitance *Q*_CT_ describe the rate of resistance and the electrochemical redox reactions that might come about at the electrode/electrolyte interface. The constant phase element *Q*_L_ and leakage resistance *R*_L_ are associated with electrical double layer capacitance. Warburg impedance *W*, associated to the transition from high frequency to low frequency is related to diffusion of the ions in the cell.^44^

Stability test is one of the main factors used to determine the performance of a device. The capacity retention and coulombic efficiency of the device were measured as a function of cycle numbers at a specific current of 5 A g^−1^ over 5000 cycles as demonstrated in Fig. 6(f). From the figure, the device's capacity was noticed to be fluctuating but became very stable at about 2000 cycles and beyond. This is due to the electrode material's lack of enough wettability at the initial cycles. The device could retain an 89% of its initial capacity as well as a coulombic efficiency of approximately 94% over 5000 cycles, suggesting the material's potential for electrochemical devices applications.

**Fig. 7:**
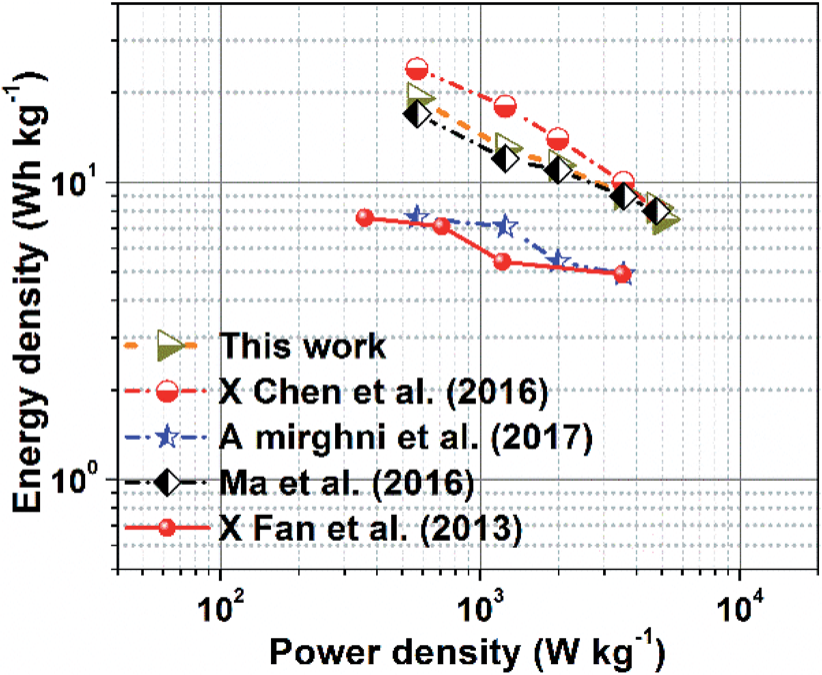
Ragone plots of NaNi_4_(PO_4_)_3_/GF//AC and reported bimetallic phosphate based in literature.

Fig. 7 presents a Ragone plot which demonstrates the position of the device in this work in terms of energy density and power densities in comparison to some recently published bi-metallic phosphate-based devices from the literature. The NaNi_4_(PO_4_)_3_/GF//AC hybrid device delivered a highest energy and power density of 19.5 W h kg^−1^ and 570 W kg^−1^, respectively, at a specific current of 0.5 A g^−1^ within extended cell potential of 0.0–2.0 V. These energy and power densities values were calculated according to eqn (4) and (5) below:4
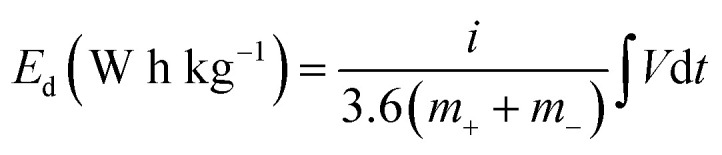
5
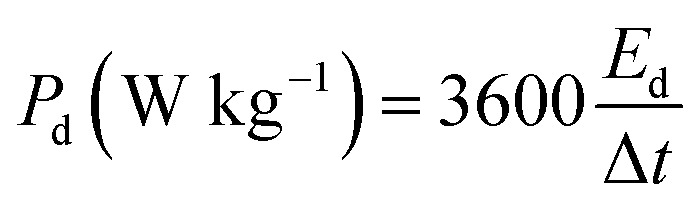
where *i* represent applied current (mA), *m* represent the total mass of the active material (mg), 
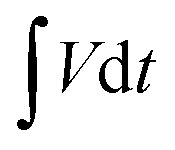
 is the area under the discharge curve of the device, and Δ*t* is the discharge time (s).

The results presented in Table 1 show the significance of our work in terms of improved energy and power densities as well as extended cell operating potential compared to similar work recently reported in the literature.

**Table 1:** Performance comparison of hybrid NaNi_4_(PO_4_)_3_/GF//AC and some phosphate/oxide-based materials from the literature

Hybrid device	Electrolyte	Specific current (A g^−1^)	*E*_d_ (W h kg^−1^)	*P*_d_ (W kg^−1^)	Cell potential (V)	Ref.
AC//NaMn_1/3_Ni_1/3_Co_1/3_PO_4_	NaOH	0.5	15	400	1.6	45
AC//NaNiPO_4_	NaOH	1.0	20	138	1.6	25
AC//NaMnO_2_	Na_2_SO_4_	1.0	19.5	130	1.9	46
Mn_3_(PO_4_)_2_//AC	Na_2_SO_4_	0.5	16.64	400.03	1.6	47
Mn_3_(PO_4_)_2_//Mn_3_(PO_4_)_2_	KOH	0.5	17.21	399.72	1.6	47
NaNi_4_(PO_4_)_3_/GF//AC	NaNO_3_	0.5	19.5	570	2.0	This work

## 4. Conclusion

In conclusion, a hierarchical spherical morphology of sodium nickel phosphate graphene foam composite (NaNi_4_(PO_4_)_3_/GF) was synthesized through direct and simple chemical precipitation method. The material has been tested in three electrode measurement using 2 M NaNO_3_ electrolyte within working potential of 0.0–1.0 V. A specific capacity of 63.3 mA h g^−1^ was obtained at 1 A g^−1^ specific current. A hybrid device was a assembled with NaNi_4_(PO_4_)_3_/GF composite as a positive electrode and AC as a negative electrode, and was found to exhibit specific capacity of 15.1 mA h g^−1^, energy density of 19.5 W h kg^−1^ and a power density of 570 W kg^−1^ at specific current of 0.5 A g^−1^.

## Conflicts of interest

There are no conflicts to declare.

## Supplementary Material
